# The practice of intracytoplasmic sperm injection in Jordan: A clinical outcome study

**DOI:** 10.1016/j.amsu.2020.07.042

**Published:** 2020-07-28

**Authors:** Omar F. Altal, Abdelwahab J. Aleshawi, Mohammad Z. Khrais, Bouran M. Alkilany, Tasneem M. Abudalo, Ahmed H. Al Sharie, Faheem Zayed

**Affiliations:** a Department of Obstetrics & Gynecology, Faculty of Medicine, Jordan University of Science & Technology, Irbid, Jordan; b Intern, King Abdullah University Hospital, Jordan University of Science & Technology, Irbid, 22110, Jordan; c Faculty of Medicine, Jordan University of Science & Technology, Irbid, 22110, Jordan

**Keywords:** Intracytoplasmic sperm injection, Assisted reproductive technique, Gestational hypertension, Multiple gestation

## Abstract

**Objective:**

The lack of appropriate guidelines and justified practice in most developing countries is a serious limitation to efforts to qualify the services provided. In this paper, we evaluate and assess the clinical practice of an assisted reproductive technique of intracytoplasmic sperm injection (ICSI) in Jordan.

**Methods:**

Retrospectively, we identified women who achieved a successful pregnancy by ICSI over a period of ten years. Information related to the ICSI procedure, foetus abnormalities, maternal complications and delivery outcomes were included. A control group of normal pregnancies were incorporated.

**Results:**

In total, 291 cases were included as successful cases of ICSI with a success rate of 14.1%. For the control group, 466 pregnant women with normal pregnancies were included. No statistical difference was observed between both groups in foetus malformation. In normal pregnancy women, 42.1% delivered through caesarean section (CS) while 87.6% of ICSI patients underwent CS. Women with ICSI had significantly higher rates of multiple gestations, and their neonates were lower in birthweight. Women in the ICSI group tended to deliver females compared to the control group. Pregnancy-related hypertension was more commonly reported in the normal pregnancy group, while gestational diabetes, antepartum haemorrhage and preterm labour were more common in ICSI group.

**Conclusion:**

Services for the management of infertility are increasing in the Middle East. The clinical outcomes in Jordan is approaching that of other developed regions, although the success rate is lower than in other regions. Further studies and efforts should be carried out to maximize effective and successful practice in such low-income areas.

## Highlights

• The practice of ICSI needs to be justified in the developing countries.
• The success rate of ICSI is low with higher rates of CS and multiple gestation.
• Neonates for women with ICSI are born with lower birth weight.

## 1 Introduction

Since the first U.S. infant conceived using an assisted reproductive technique (ART) in 1981, many advanced technologies have been used to overcome infertility. ART includes adding the sperm and the ovum together outside the human body (*in vitro*), and the routine *in vitro* fertilization (IVF) procedure includes three steps: 1-ovarian hyperstimulation, 2-IVF and embryo culture, and 3-embryo transfer, and sometimes includes additional *in vitro* procedures (e.g. intracytoplasmic sperm injection (ICSI)) [[1], [2], [3], [4]]. Intrauterine insemination and medical ovarian stimulation are not considered ART, which must include retrieving both gametes and embryo *in vitro*. According to the CDC (The Centres for Disease Control and Prevention) data report in the United States for 2013, the most common diagnosis reported among 190773 cycles of ART was diminished ovarian reserve (18%) followed by male and female factors (17%) and male factors (16%). On the other hand, the least common diagnoses were tubal factors (6%), endometriosis (3%), and uterine factors (1%) [5,6].

ICSI has been used in a variety of challenging infertility cases. This procedure, which involves the injection of a single sperm cell directly into the ooplasm, allows bypassing zona pellucida irregularities (like zona hardening in cryopreserved oocytes), overcoming male factor problems including the presence of anti-sperm antibodies, sperm acrosome dysfunction and sperm kinetic defects, in addition to the ability of utilizing microsurgically collected gametes from azoospermic patients [7,8].

In a report from 2002, there were over 186 million couples in developing countries alone (excluding China) who were affected by infertility (both primary and secondary) [9]. When talking about role of ART in developing countries, many concerns and barriers become apparent. The main concerns are whether expensive techniques, which have a low success rate (live birth rate, 25% per cycle), can be justified in countries where poverty is still an important issue [9]. In this article, we present the practice of ICSI in one of the countries in the developing Middle East, Jordan, in order to reflect the main pros and cons and compare it to other practices in terms of clinical outcomes.

## 2 Material and methods

This study was conducted at a tertiary hospital located in Jordan. After obtaining Institutional Review Board approval (IRB), we retrospectively identified those patients who were referred to the fertility service for ICSI over a period of ten years. The following information was obtained: demographics (e.g. age), ICSI-related factors, foetus abnormalities and pregnancy and maternal complications. In addition, the mode of delivery and outcome of pregnancy were included. A control of group of normal pregnancies were also retrieved. Females in the control group were chosen as healthy candidates without any significant gynaecological surgical history or medical history which could affect their pregnancy course. This study was conducted according to the STROCSS 2019 guideline [10].

The included group was comprised of patients who achieved successful pregnancies following ICSI. ICSI-related factors include number of oocytes retrieved, number of oocytes fertilized, types of male specimen, results of sperm analysis for their husbands, and numbers and grades of embryo transferred. The types male specimen was fresh ejaculate, fresh testicular biopsy, frozen ejaculate and frozen testicular biopsy. The included sperm analysis tests were sperm count (a normal sperm count is at least 15 million sperm per millilitre of semen), sperm motility (a normal semen motility is when grade a and b are more than >32%), volume (a normal value is more than 1.5 mL) and morphology.

The foetus abnormalities include nuchal translucency in the first trimester, detailed second trimester ultrasound scanning for organ abnormalities and malformations, and small for gestational age (SGA). Maternal complications included gestational hypertension, gestational diabetes, preterm labour, premature rupture of membrane and antepartum haemorrhage. The outcome of pregnancy was measured by number of gestations, the gender of neonates and birth weight.

The control group included pregnant women who conceived without ART. The same measures related to foetus abnormalities and pregnancy and maternal complications, mode of delivery, and outcome of pregnancy were included. The IVF team who was responsible for admission consisted of consultants in obstetrics and infertility medicine who followed the same guidelines.

Data were entered into a spreadsheet. Statistical analyses were performed using IBM SPSS Statistics Software (v.21), 2012. Data were presented as frequency distributions for categorical variables and mean ± standard error of the mean for continuous variables. Data were tested at a significance level of 0.05%. Pearson χ^2^ test was used to investigate the significance of the association between categorical variables, while student's *t*-test and ANOVA were applied to examine the significance level for continuous normally distributed variables. The normality of the distribution of data was tested using the Kolmogorov-Smirnov test. If a significant (*P* < 0.05) relationship was found, then a *posthoc* residual analysis for categorical variables and a Fisher's least significant difference test for continuous variables were applied to determine the exact significance between groups for each variable. Multiple linear and multiple logistic regression analyses were utilized to study the multiple effects of different variables.

## 3 Results

### 3.1 General characteristics

Two hundred and ninety-one patients were included in this study as successful cases of ICSI out of 2059 case of total ICSI, with a success rate of 14.1%. Also, 466 pregnant women were included as a control group of normal pregnancy. The mean age was 29.7 years. Two hundred and fifty-five of those who had ICSI underwent caesarean section (CS) compared to 36 (12.4%) who had normal vaginal delivery (NVD). The mean birthweight for both groups was 2.9 kg.

For men who were included in the study and underwent ICSI, the mean sperm count was 41.3 million/mL, the median was 25.0 million/mL, and 101 (34.7%) men had a sperm count below normal. Also, the mean for sperm motility was 41.1%, the median was 43% and 99 had abnormal motility (34%). The fresh ejaculate constitutes 88.3% of male specimens, while 2.7% had a fresh testicular biopsy, 7.9% had a frozen testicular biopsy and 1.1% had frozen ejaculate. Also, the mean number of retrieved oocytes was 9.1, the median was 8.0 oocytes, one retrieved oocyte was the smallest number, and 27 oocytes were retrieved as the largest number. However, the mean number for fertilized oocytes was six, the median was five, the minimal was one oocyte and the maximum was 25 oocytes. The mean for transferred embryos was 2.8, the median was three, the minimum was one and the maximum was three, as presented in Table 1.

**Table 1:** General characteristics of the included sample.

Variables	Number	Percent (%)
	Mean ± SE	
**Age**	29.7 ± 0.2	
**Type of pregnancy**		
ICSI	291	38.4
Normal pregnancy	466	61.6
**Oocyte retrieved**	9.1 ± 0.3	
**Oocyte fertilized**	5.9 ± 0.2	
**Embryo transferred**	2.8 ± 0.0	
**Embryo grade**		
Grade 1	217	74.6
Grade 2	61	21.0
Morula	13	4.5
**Nuchal translucency**		
Normal	751	99.2
Abnormal	6	0.8
**Second detailed US**		
Normal	736	97.2
Abnormal	21	2.8
**SGA**		
Yes	128	16.9
No	629	83.1
**Maternal pregnancy complications**		
No	600	79.3
HTN	25	3.3
Gestational diabetes	12	1.6
Preterm labour	103	13.6
PROM	1	0.1
Antepartum haemorrhage	15	2.0
Anaemia	1	0.1
**Mode of delivery**		
Vaginal	306	40.4
CS	451	59.6
**Gestational number**		
Single pregnancy	668	88.2
Twins	76	10.0
Triplet	13	1.7
**Neonates gender (*n*** = **859 neonate)**		
Male	429	49.9
Female	430	50.1
**Birthweight (kg)**	2.9 ± 0.0	
**Foetus abnormality**		
No	736	97.2
Head	4	0.5
Cardiac	7	0.9
Abdominal	1	0.1
Skeletal	2	0.3
Urogenital	1	0.1
Two abnormalities	4	0.5
Three abnormalities	2	0.3
**Type of male semen**		
Fresh ejaculate	257	88.3
Fresh testicular biopsy	8	2.7
Frozen testicular biopsy	23	7.9
Frozen ejaculate	3	1.0
**Sperm count**	41.3 ± 2.9	
**Sperm motility**	41.1 ± 1.4	

**Abbreviations**: **ICSI**: intracytoplasmic sperm injection; **US**: ultrasound; **SGA**: small for gestational age; **HTN**: hypertension; **PROM**: premature rupture of membranes; **CS**: caesarean section; **SE**: standard error of the mean.

### 3.2 Outcomes of ICSI

From women with normal pregnancy; 196 (42.1%) delivered through CS while 255 (87.6%) of ICSI patients underwent CS (*P* = 0.00). Also, 287 (98.6%) of those who underwent ICSI had normal nuchal translucency and 464 (99.6%) in normal pregnancy (P > 0.05).

The majority of patients had a second trimester detailed ultrasound without malformations and without statistical difference; in seven cases in ICSI and 14 cases of normal pregnancy, the ultrasound detected abnormalities. Regarding foetus abnormalities, ICSI patients’ foetuses did not shown any head, abdominal, urogenital or multiorgan-abnormalities, whereas the products of the normal pregnancies showed slightly different results, including four foetuses (0.9%) which had head abnormalities, one (0.2) which had an abdominal abnormality, one (0.2) which had an urogenital abnormality, four (0.9%) which had two abnormalities and two (0.4%) which had three-abnormalities On the other hand, foetuses from the ICSI procedure showed a higher percentage of cardiac abnormalities, represented in four foetuses (1.4%), whereas normal pregnancy foetuses showed only three (0.6%) such abnormalities. Also, regarding skeletal abnormalities; two (0.7%) were seen in ICSI foetuses, but there were none in normal pregnancy foetuses.

Regarding SGA, of those foetuses that were products of ICSI, 87 were small for their gestational age (27.9%). This number was much lower in normal pregnancies, which had 41 foetuses (8.8%) with SGA (P = 0.00). This related to the number of gestations for each group, which will be presented later.

Part of our study included the maternal pregnancy complications in both groups. In general, ICSI group had a higher number of pregnancy complications. There were 207 (71.7%) in ICSI group and 393 (84.3%) in normal pregnancy group without complications (*P* = 0.001). However, interestingly, 3 mothers (1.0%) had gestational hypertension from the ICSI group, whereas the normal pregnancy group showed 22 cases of gestational hypertension (4.7%) (*P* = 0.01). Also, eight (2.7%) had DM from the ICSI group, whereas four (0.9%) had DM in normal pregnancy (*P* = 0.03). Moreover, 62 (21.3%) of the ICSI patients had preterm labour versus 41 (8.8%) in the normal pregnancy group (*P* = 0.001), as shown in Table 2. Furthermore, one patient had premature rupture of the membrane in normal pregnancy, while ICSI did not have any cases of such a complication (*P* > 0.05). In addition, antepartum haemorrhage had the percentage of 3.4% (10 patients in ICSI group) and five patients (1.1%) in normal pregnancy (*P* = 0.027). One patient (0.2%) had anaemia in normal pregnancy, but none were seen in the ICSI group (*P* > 0.05).

**Table 2:** Outcome of ICSI compared to normal pregnancies.

Variables	ICSI *n* (% from ICSI)	Normal pregnancy *n* (% from normal pregnancy)	*P*-value
**Age (years, mean** ± **SE)**	30.0 ± 0.3	29.6 ± 0.3	NS
**Nuchal translucency**			
Normal	287 (98.6)	464 (99.6)	NS
Abnormal	4 (1.4)	2 (0.4)	
**Second detailed US**			
Normal	284 (97.6)	452 (97.0)	NS
Abnormal	7 (2.4)	14 (3.0)	
**SGA**			
yes	87 (27.9)	41 (8.8)	0.000
No	204 (70.1)	425 (91.2)	
**Foetus abnormality**			
No	285 (97.9)	451 (96.8)	
Head	0 (0.0)	4 (0.9)	
Cardiac	4 (1.4)	3 (0.6)	
Abdominal	0 (0.0)	1 (0.2)	
Skeletal	2 (0.7)	0 (0.0)	NS
Urogenital	0 (0.0)	1 (0.2)	
Two abnormalities	0 (0.0)	4 (0.9)	
Three abnormalities	0 (0.0)	2 (0.4)	
**Maternal pregnancy complications**			
No	207 (71.7)	393 (84.3)↑	
HTN	3 (1.0)	22 (4.7)↑↑	
Gestational diabetes	8 (2.7)↑	4 (0.9)	
Preterm labour	62 (21.3)↑	41 (8.8)	0.00
PROM	0 (0.0)	1 (0.2)	
Antepartum haemorrhage	10 (3.4)↑	5 (1.1)	
anaemia	0 (0.0)	1 (0.2)	
**Mode of delivery**			
Vaginal	36 (12.4)	270 (57.9)	0.000
CS	255 (87.6)	196 (42.1)	
**Number of gestations**			
Single pregnancy	212 (72.9)	456 (97.9)	
Twins	66 (22.7)	10 (2.1)	0.00
Triplets	13 (4.5)	0 (0.0)	
**Gender of neonates**			
Male	171 (58.8)	258 (55.4)	0.00
Female	212 (72.9)↑↑	218 (46.8)↓	
**Birthweight (kg, mean** ± **SE)**	2.7 ± 0.04	3.1 ± 0.03	0.00

**Abbreviations**: **ICSI**: intracytoplasmic sperm injection; **US**: ultrasound; **SGA**: small for gestational age; **HTN**: hypertension; **PROM**: premature rupture of membranes; **CS**: caesarean section; **SE**: standard error of the mean. **↑** Significantly higher. **↓** Significantly lower.

The ICSI group had significantly more multiple gestations. In ICSI, there were 13 triplets and 66 twins, compared to no triplets and 10 twins in the normal pregnancy group. In addition, ICSI produced significantly more female neonates than did normal pregnancies. As a result of the increased number of multiple gestation pregnancies in ICSI, the birthweight of the neonates was significantly lower than in the normal pregnancies. The results are summarized in Table 2.

## 4 Discussion

To the best of our knowledge, this is an updated study that presents the outcome of the practice of ICSI in Jordan. The success rate is relatively low (14.1%). Additionally, more SGA babies were found in the ICSI group and the birthweight of the babies was lower. This is attributed to the higher numbers of multiple gestations in the ICSI group. Also, as a result, the ICSI group had higher rates of CS. However, the presence of malformations was relatively low compared to the normal pregnancies. Females constituted the majority of the neonates in the ICSI group. Gestational diabetes, preterm labour and antepartum haemorrhage were more commonly encountered in the ICSI group. Interestingly, gestational hypertension was developed more often in the normal pregnancy group.

In 2010, among 4,046,553 infants born in the U.S, a total number of 59,119 (1.5%) were conceived with ART procedures. A report by the CDC for ART procedures outcomes in the United States was compared to the outcomes of all infants born there in 2010. Regarding singleton and multiple births, 46.4% of ART infants were multiples compared with only 3.4% of all infants. A mean of two embryos was transferred per cycle among all age groups, which is explained both by providers and patients being willing to maximize the success rates of having a live birth in a single procedure. Regarding birthweight, low birthweight (<2500 g), moderate low birthweight (1500–2500 g) and very low birthweight (<1500 g) were higher among infants conceived with ART (31.6% were low birthweight) than among all infants (8.2% were low birthweight) [6].

A controlled study comparing chromosomal abnormalities and major malformations between infants conceived by IVF or ICSI (group 1) to naturally conceived infants (group 2) between 1993 and 1997 in Western Australia demonstrated that chromosomal and musculoskeletal defects were two-folds higher in group 1 compared to group 2. This study was controlled for parental factors such as maternal age and parity, the gender of the infant and correlation between siblings. Limitations in the previous study arose from the difficulty in excluding the effects of ART procedures from the underlying subfertility as the cause of the abnormalities [11].

In a systematic review of worldwide trends in ART between 2004 and 2013, a regional variation in the outcome was observed. New practices such as Single Embryo Transfer (SET) and the utilization of frozen-thawed embryos increased worldwide over the study period. This trend, in addition to minimal ovarian stimulation protocols, resulted in a decline in fresh cycle live birth rates in Japan. Embryo selection, including blastocyst-stage transfer, cryopreservation of all embryos and subsequent frozen-thawed transfer, and the utilization of SET, was integrated into ART. However, these may have negatively affected outcomes in poor prognosis patients [[12], [13], [14], [15], [16], [17], [18]]. About 72% of failed ART procedures are due to implantation failure, which is a complex process requiring both a healthy blastocyst and a functionally receptive endometrium [19].

In a study by Xiong et al., in 2017, the authors stated that the risk of gestational hypertension in pregnancy has a certain correlation with the ICSI fertilization technology [20]. In addition, many studies supported in their results that ART pregnancy has been reported as a factor linked to an increased risk for hypertension [[21], [22], [23], [24]]. As reported, the exact aetiology is still unknown. However, a postulated explanation is that reduced reproductive capacity, manifested as infertility, might be involved in the pathogenesis of hypertension. This theory supports the idea that preconception maternal factors such as age, rather than the type of ART procedure, are linked to the occurrence of hypertension in those studies [21]. Another possibility is that hormonal pre-treatment or the ART procedure directly affects the incidence of hypertension [21]. Nevertheless, interestingly, our results differ from the aforementioned studies in that gestational hypertension was reported much more often in normal pregnancies. This could be explained by the fact that women with endothelial dysfunction, who are prone to develop pregnancy-related hypertensive disorder, did not conceive at all, even after ICSI, and were not included in the study. However, unfortunately, data on these failed cases were not available.

Increased multiple gestations after ICSI is an important factor in increasing obstetric haemorrhages such as antepartum haemorrhage. Placenta previa is also known to be more often reported with single birth deliveries after ART than after normal pregnancies, though without known mechanisms [25]. Smithers et al. reported a high occurrence rate of antepartum haemorrhage in IVF twin pregnancies [26]. They explained this difference as a consequence of embryo transfer through the vagina and cervix, compared with *in-vivo* conception and implantation via the uterine tube [26]. Our results are consistent with these studies.

In the current work, we provide a retrospective, comprehensive and systematic update on the practice of intracytoplasmic sperm injection in Jordan in an outcome clinical study fashion. This study had an important limitation due to its retrospective nature and the loss failed pregnancy cases, but such an update could be useful for representing what intracytoplasmic sperm injection parameters looks like in a developing country such as Jordan.

## 5 Conclusion

The treatment of infertility possess an extremely important value in the Middle East, because a women's social status and self-esteem are associated with her ability to have children. Accordingly, it is not surprising that there is an increased demand for services in this area. The lack of clear guidelines and regulations in most developing countries is a serious limitation to improving the quality of ART and decreasing its side effects. In Jordan, ICSI services are growing and improving, although the success rate is less than that of other regions. However, many of the clinical parameters are similar. Further studies in developing countries are encouraged in order to encounter the most effective and successful practices for such low-income areas.

## Ethical approval

Institutional approval was obtained from the Institutional Review Board at Jordan University of Science and Technology.

This study has been performed in accordance with the ethical standards laid down in the 1964 Declaration of Helsinki and its later amendment. This research has obtained ethical approval from Research and Ethics Committee, at Jordan University of Science and Technology and King Abdullah University Hospital, Irbid, Jordan.

## Sources of funding

No funding

## Author contribution

All authors contributed significantly and in agreement with the content of the article. All authors were involved in project design, data collection, analysis, statistical analysis, data interpretation and writing the manuscript. All authors presented substantial contributions to the article and participated of correction and final approval of the version to be submitted.

## Trial registry number

UIN: researchregistry5618.

## Guarantor

Omar Altal.

## Consent

Written informed consent was waived due to the retrospective nature of the study.

## Funding

The authors have not declared any grant for this work from any funding authority.

## Disclosure

The authors have no financial ties or conflicts of interest to disclose.

## Consent for publication

Not required.

## Provenance and peer review

Not commissioned, externally peer reviewed.

## Declaration of competing interest

The authors declare that they have no competing interests.
